# Dealing with symptoms in the general population: lessons learned from the Danish Symptom Cohort

**DOI:** 10.3399/bjgp22X720713

**Published:** 2022-10-30

**Authors:** Kirubakaran Balasubramaniam, Sanne Rasmussen, Peter Fentz Haastrup, Lisa Maria Sele Sætre, Jens Søndergaard, Dorte Ejg Jarbøl

**Affiliations:** Research Unit of General Practice, Department of Public Health, University of Southern Denmark, Denmark.; Research Unit of General Practice, Department of Public Health, University of Southern Denmark, Denmark.; Research Unit of General Practice, Department of Public Health, University of Southern Denmark, Denmark.; Research Unit of General Practice, Department of Public Health, University of Southern Denmark, Denmark.; Research Unit of General Practice, Department of Public Health, University of Southern Denmark, Denmark.; Research Unit of General Practice, Department of Public Health, University of Southern Denmark, Denmark.

Every day many people experience symptoms ranging from barely noticeable sensations to disturbing discomforts. The causes vary from normal physiological processes and self-limiting benign conditions to life-threatening diseases. Symptom experiences do not occur as stand-alone events but are influenced by an interchange of biological, psychological, and cultural factors, leading to various ways of interpreting and managing symptoms.^1^ Consequently, many symptoms are managed privately without consulting the healthcare system;^2^ however, some symptoms should lead to further investigation as they are alarming from a healthcare perspective and timely investigation is warranted.^3^^,^^4^ GPs play an important role in helping patients interpret symptoms;^5^ however, the healthcare-seeking process is not simple, and numerous factors, including previous experiences, social relations, and support, may contribute in deciding whether or not to seek health care.^6^

To elucidate how symptoms are interpreted and managed in the general population, we initiated the Danish Symptom Cohort (DaSC), a nationwide web-based survey. In 2012, we invited 100 000 randomly selected individuals aged >20 years to complete a questionnaire covering 44 predefined symptoms comprising both cancer symptoms as well as common symptoms, for example, back pain, urinary incontinence, and tiredness. If responders confirmed a symptom experience, follow-up questions were asked concerning onset, influence on daily activities, concerns about the symptom, whether the responder had consulted the GP regarding the symptom, and considerations about contacting the GP with the symptom in question. Responders were also asked about smoking status, alcohol intake, and physical activity. For the invitees, socioeconomic status (education, income, cohabitation status, ethnicity, and labour market affiliation) were then collected from national registers^7^^–^^11^ and linked to survey data.

In this editorial we summarise the findings from the DaSC studies, highlighting the lessons learned but also pointing to what needs to be further explored in the 10-year follow-up questionnaire, DaSC II, which was distributed in May 2022.

The 34 current publications deriving from the DaSC are available online,^12^ with details of the results and contextualisation of findings within the existing literature.

## FREQUENCY OF SYMPTOMS AND GP CONTACT

The survey response rate was 52.2%. The median age was 51 years (interquartile range 38–65 years), and responders were fairly representative of the study sample.

About 9 out of 10 individuals reported at least one of the 44 predefined symptoms within the preceding 4 weeks. Prevalence estimates varied from 49.4% ( *n* = 24 537) reporting tiredness to 0.1% ( *n* = 54) reporting blood in vomit. The mean number of reported symptoms was 5.4 (males 4.8; females 6.0) and 37% contacted their GP about at least one symptom.

The symptoms leading to the highest proportion of GP contacts were relatively infrequently reported symptoms with visible signs, such as blood in the urine, blood in semen, and coughing up blood. For potentially more serious symptoms such as unintentional weight loss, rectal bleeding, or post-menopausal bleeding, no more than one-third reported having contacted their GP.

The DaSC study results increase understanding of the complexity of symptom experiences and healthcare-seeking behaviour in the general Danish population, which in many ways is comparable to the UK and other western European populations. As would be expected, most symptoms are not presented to the GP. Many bodily sensations such as tiredness are common and seldomly on their own a sign of disease. Thus, it seems appropriate that the public does not present as patients with such a symptom. However, it is disturbing that some symptoms indicative of potentially serious diseases, such as rectal bleeding, only infrequently lead to GP contact, and that to some extent such symptoms are perceived as too embarrassing to discuss with the GP. Likewise, it is thought-provoking that benign symptoms, such as urinary incontinence and erectile dysfunction, rarely lead to GP contact even though the symptoms are reported as bothersome. These symptoms are common and often treatable. Both GPs and patient organisations could be important facilitators in disseminating knowledge about symptoms and treatment options to the general population, thereby decreasing feelings of taboo and shame, and improving quality of life for many people.

## SOCIODEMOGRAPHIC DIFFERENCES IN GP CONTACT

While females have been observed to contact GPs more frequent than males, our findings provide nuance to this observation by revealing that females experience more symptoms than males. For almost two-thirds of the symptoms reported, no differences were found between sexes concerning the proportion of GP contacts. However, females contacted GPs more often with headache, repeated vomiting, tiredness, and lack of energy, whereas males contacted GPs more often with urogenital symptoms, for example, difficulty emptying the bladder, frequent urination, and night-time urination.

Generally, we found that older individuals contacted their GP more often when experiencing symptoms. A possible explanation is the finding that younger individuals more often reported barriers to contacting the GP. Common barriers were worrying about wasting the doctor’s time, being worried about what the doctor might find, and being too busy to contact the doctor, despite reporting bothersome symptoms or potential cancer symptoms. Many responders of all ages hesitated to contact their GP about urogenital and bowel symptoms, and moreover reported barriers like embarrassment.

Among lifestyle factors, individuals who smoked reported a different GP contact pattern. For respiratory symptoms, individuals who smoked reported a higher frequency of symptoms but a lower likelihood of contacting their GP. Furthermore, individuals who smoked also reported more barriers to GP contact with respiratory symptoms than individuals who had never smoked. Individuals out of the workforce, comprising disability pension and unemployment benefit, were also more likely to report barriers such as worrying about what the doctor might find and worrying about wasting the doctor’s time regarding respiratory and urogenital symptoms. For some, this might be due to self-blame or stigma, but the normalisation of symptoms or a lack of knowledge about treatment options might also act as a barrier. Increased awareness in general practice or organisational initiatives could include a special focus on GP accessibility to improve outcomes for selected groups of patients who may avoid health care.

## INVOLVEMENT OF PERSONAL AND PROFESSIONAL RELATIONS

For both potential cancer symptoms and several bothersome symptoms, responders who had discussed the symptoms with family or friends were more likely to contact the GP. The most frequently involved relation was the spouse or partner, but almost one-third did not involve any personal relation. Concern about the symptom and/ or the symptom affecting daily activity increased the likelihood of GP contact.

Moreover, we found that individuals without an available social network were more prone to contact their GP. This highlights the importance of GPs and other healthcare professionals being aware of patients’ networks, to ensure a safety net and facilitate open-minded dialogue with at-risk individuals.

## PERSPECTIVES OF THE DaSC

Factors, such as organisation of general practice and previous experiences with general practice and the healthcare system likely also affect healthcare-seeking behaviour, but the DaSC questionnaire did not address such factors. In May 2022, we established the DaSC II and distributed a new questionnaire to the responders from 2012, and to 100 000 new randomly selected Danish adults. The DaSC II questionnaire investigates how the organisation of general practice, previous experiences with GP contacts, and health literacy may influence symptom experiences and GP contact. Current medical practice is characterised by a focus on risk reduction and early detection of diseases, which, combined with developments in biomedical knowledge and diagnostic technologies, may have expanded the pool of potential symptoms. Bodily changes, feelings, or sensations may be designated as symptoms and potential signs of disease more often. One hypothesis is, therefore, that we will see a higher frequency of GP contacts in 2022.

The lessons learned from the DaSC have already been disseminated directly to GPs and other healthcare professionals, for example, through postgraduate training courses, increasing awareness about individuals hesitating from healthcare seeking. By gaining even more knowledge about symptoms and healthcare-seeking behaviour in the general population through DaSC II, we aim to contribute to an improved and more accessible healthcare system, enhancing the chances for timely diagnosis and improved prognosis in the future.
